# The Importance of Ambient Temperature to Growth and the Induction of Flowering

**DOI:** 10.3389/fpls.2016.01266

**Published:** 2016-08-23

**Authors:** C. R. McClung, Ping Lou, Victor Hermand, Jin A. Kim

**Affiliations:** ^1^Department of Biological Sciences, Dartmouth College, Hanover, NHUSA; ^2^Department of Agricultural Biotechnology, National Academy of Agricultural Science, Rural Development Administration, Jeonju-siSouth Korea

**Keywords:** circadian clock, circadian rhythms, photoperiodic flowering, flower induction, thermoresponsive flowering

## Abstract

Plant development is exquisitely sensitive to the environment. Light quantity, quality, and duration (photoperiod) have profound effects on vegetative morphology and flowering time. Recent studies have demonstrated that ambient temperature is a similarly potent stimulus influencing morphology and flowering. In *Arabidopsis*, ambient temperatures that are high, but not so high as to induce a heat stress response, confer morphological changes that resemble the shade avoidance syndrome. Similarly, these high but not stressful temperatures can accelerate flowering under short day conditions as effectively as exposure to long days. Photoperiodic flowering entails a series of external coincidences, in which environmental cycles of light and dark must coincide with an internal cycle in gene expression established by the endogenous circadian clock. It is evident that a similar model of external coincidence applies to the effects of elevated ambient temperature on both vegetative morphology and the vegetative to reproductive transition. Further study is imperative, because global warming is predicted to have major effects on the performance and distribution of wild species and strong adverse effects on crop yields. It is critical to understand temperature perception and response at a mechanistic level and to integrate this knowledge with our understanding of other environmental responses, including biotic and abiotic stresses, in order to improve crop production sufficiently to sustainably feed an expanding world population.

## Introduction

Plant development is highly sensitive to the environment. For example, light dramatically affects plant morphology (Arsovski et al., 2012). When grown in the dark, dicot seedlings become etiolated, develop elongated hypocotyls, and are pale because chloroplast formation and chlorophyll biosynthesis requires exposure to light. In contrast, seedlings grown in the light undergo photomorphogenesis, exhibiting short embryonic stems and expanded green cotyledons. Light quality also has a profound influence on plant morphology. Shading, in which the ratio of red to far-red light is decreased, induces a suite of morphological changes that includes the elongation of hypocotyls and petioles and upward (hyponastic) growth of the petioles and leaves to yield an open rosette (Casal, 2012). In addition, the relative duration of light and dark during the day, photoperiod, has a major influence on the transition to flowering (Song et al., 2015).

Plant morphology and reproductive development are also strongly influenced by temperature (Wigge, 2013; Quint et al., 2016). Ambient temperatures that are high, yet insufficient to cause heat stress, induce a suite of morphological changes that are collectively termed thermomorphogenesis. In *Arabidopsis*, growth at 27°C results in elongated hypocotyls and petioles and other morphological changes that are reminiscent of the response to shade. In addition, elevated temperature accelerates flowering, especially in short days that are non-inductive in *Arabidopsis* grown at lower ambient temperatures (e.g., 15–20°C).

In this mini review, we will consider the similarities and differences in the thermoresponsiveness of growth and flowering, with an emphasis on *Arabidopsis*, where our mechanistic understanding is greatest.

## Thermomorphogenesis

One of the first manifestations of thermomorphogenesis, the suite of responses in growth to elevated temperature, is increased elongation of the hypocotyl. The similarity of thermomorphogenesis to responses to shading suggested common underlying mechanisms. PHYTOCHROME INTERACTING FACTOR4 (PIF4) and PIF5, basic helix-loop-helix (bHLH) transcription factors that are key components of phytochrome signaling with central roles in photomorphogenesis (Leivar and Monte, 2014), also play pivotal roles in thermomorphogenesis (Wigge, 2013; Quint et al., 2016). Loss of PIF4 function attenuates hypocotyl elongation at elevated temperature (Koini et al., 2009). Similarly, mutants that disrupt auxin signaling block thermoresponsive hypocotyl elongation (Gray et al., 1998). PIF4 interacts with BRASSINAZOLE-RESISTANT1 to regulate many genes associated with growth regulation (Oh et al., 2012), integrating multiple hormone (auxin, brassinolide, gibberellin, and cytokinin) signaling pathways in the growth response.

Expression of PIF4 and PIF5 is tightly regulated at both transcriptional and post-transcriptional levels. *PIF4* and *PIF5* transcription and mRNA accumulation are regulated by the circadian clock (Nozue et al., 2007; Niwa et al., 2009; Kunihiro et al., 2011; Nusinow et al., 2011). During the light and early evening, *PIF4* transcription is repressed by ELONGATED HYPOCOTYL5 (HY5) and the evening complex (EC) as well as by additional transcriptional repressors (Lee et al., 2007; Toledo-Ortiz et al., 2014; Quint et al., 2016). Photoperiod affects *PIF4* expression, with transcripts accumulating during the night in short days but only at about dawn in long days. This permits increased PIF4 accumulation and greater hypocotyl elongation in short days due to the greater stability and activity of PIF4 in the dark (Quint et al., 2016). DE-ETIOLATED1 (DET1) plays a role in this stabilization of PIF4 (Dong et al., 2014; Shi et al., 2015). Although DET1 has not been shown to directly contribute to PIF4 accumulation at elevated temperatures, *det1* mutants are impaired in thermoresponsive hypocotyl growth (Delker et al., 2014). Thus, PIF4 plays a central role in integrating photoperiodic and circadian clock control of hormone signaling into the growth response through the external coincidence of clock and photoperiod regulated PIF4 expression with environmentally imposed dark (Nomoto et al., 2012b). Similarly, in long days at elevated temperature PIF4 accumulates earlier in the dark, again providing an example of external coincidence of clock-, photoperiod-, and temperature-regulated *PIF4* expression with environmentally imposed dark (Nomoto et al., 2012a).

Quantitative trait locus (QTL) mapping with *Arabidopsis* natural accessions revealed variation in thermoresponsive hypocotyl growth and implicated the EC components, *EARLY FLOWERING3 (ELF3)* and *LUX ARRHYTHMO* (*LUX*), as well as *PHYB*. The *elf3-1* loss of function mutant exhibits enhanced growth under control temperatures and does not increase growth at high temperature, coinciding with elevated *PIF4* levels under both conditions. These mutants also lose the high temperature induction of *LUX* expression suggesting that ELF3 is required for this rapid thermoresponsiveness (Box et al., 2015). Natural allelic variation in *ELF3* also alters the hypocotyl elongation response to shading; QTL mapping in an *Arabidopsis* Bay-0 x Sha recombinant inbred line (RIL) population revealed that the Bay-0 *ELF3* allele confers longer period and greater response to shade than the Sha allele (Jiménez-Gómez et al., 2010; Coluccio et al., 2011). Loss of *ELF3* also disrupts rhythmic root growth rates under diurnal and free running conditions (Yazdanbakhsh et al., 2011).

## Flowering Time in *Arabidopsis*

*Arabidopsis* has at least four flowering pathways: autonomous, vernalization, gibberellic acid (GA), and photoperiodic (Simpson and Dean, 2002; Amasino and Michaels, 2010). Recent evidence strongly supports a fifth, thermoresponsive, pathway (Capovilla et al., 2015). The autonomous pathway induces flowering in an environmentally (temperature and photoperiod) insensitive fashion. However, flowering is sensitive to environmental conditions, particularly to photoperiod and temperature. In *Arabidopsis*, a facultative long day plant, flowering is accelerated both in long days and at elevated temperatures. In addition, many accessions require vernalization, an extended period of cold temperature that mimics winter, in order to flower.

## Vernalization

**FIGURE 1:**
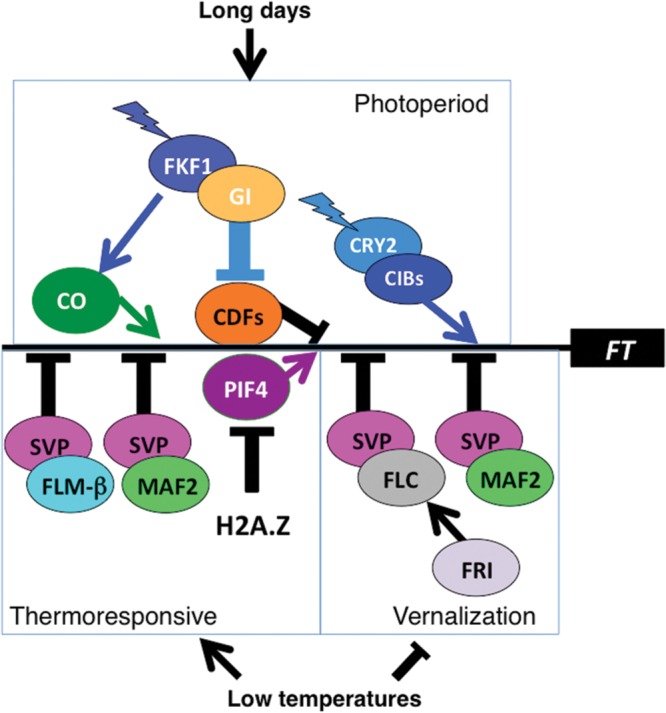
**Photoperiod and thermoresponsive pathways regulate expression of *FLOWERING LOCUS T* (*FT*).** Positive regulators of *FT* expression are indicated by white lettering and negative regulators by black lettering. The thin black horizontal line represents the FT promoter but only to indicate that all these regulatory inputs converge on the FT promoter, without depicting the number or spatial arrangement of binding sites within the promoter. Jagged arrows indicate blue light input in the late afternoon of long days, conferring photoperiod sensitivity in examples of external coincidence of light with a photosensitive phase defined by the circadian clock. Abundance of SVP (SHORT VEGETATIVE PHASE)/MAF2 (MADS AFFECTING FLOWERING 2) and SVP/FLM (FLOWERING LOCUS M)-β complexes declines with increasing temperatures. Additional complexes of SVP with other MAFs are formed but have been omitted for simplicity. Incorporation of the H2A.Z variant, which reduces access of transcriptional activators to the *FT* promoter, declines with increasing temperature. Similarly, after exposure to low temperature, *FLC* (*FLOWERING LOCUS C)* and *MAF2* expression is reduced allowing plants requiring vernalization to flower.

Much is known about vernalization in *Arabidopsis* (Kim et al., 2009; Sheldon et al., 2009; Song et al., 2012; Berry and Dean, 2015; Hepworth and Dean, 2015). Two critical components include *FRIGIDA* (*FRI*), an inducer of the flowering repressor, *FLOWERING LOCUS C* (*FLC*). FLC complexes with SHORT VEGETATIVE PHASE (SVP) to form a potent transcriptional repressor of floral inducers, including *FLOWERING LOCUS T* (*FT*), *FD*, and *SUPPRESSOR OF CONSTANS 1 (SOC1)* (**Figure 1**) (Amasino, 2010). The expression of *FLC*, which encodes a MADS domain transcriptional repressor, is progressively downregulated in response to chromatin changes resulting from prolonged (weeks to months) cold. Loss of function of either *FRI* or *FLC* eliminates the vernalization requirement, permitting accelerated flowering and a summer annual lifecycle, whereas accessions with functional *FRI* and *FLC* genes have a vernalization requirement and a winter annual habit (Gazzani et al., 2003; Song et al., 2012). A second transcriptional repressor closely related to FLC, MADS AFFECTING FLOWERING 2 (MAF2), is more slowly downregulated in response to vernalization than is FLC, and prevents premature vernalization in response to brief cold spells, although loss of MAF2 function does not eliminate the vernalization requirement (Ratcliffe et al., 2003). Like FLC, MAF2 also interacts with SVP; multiple tetrameric complexes, such as FLC-SVP-MAF3-MAF4 and SVP-FLM (FLOWERING LOCUS M)-MAF2-MAF4 have been postulated (Gu et al., 2013b; Airoldi et al., 2015).

## Thermoresponsive Flowering

The transition to flowering is influenced by moderate changes in ambient temperature. Genome-wide association (GWAS) and QTL studies in *Arabidopsis* indicate a complex architecture of natural variation in thermal responses (Sanchez-Bermejo et al., 2015). A growth promoting temperature change from 23 to 27°C is as effective at inducing flowering as the transfer from non-inductive (8-h) short days to inductive (16-h) long days (Balasubramanian et al., 2006). Although, thermoresponsive flowering is not as well understood as vernalization, evidence supports a number of independent thermoresponsive pathways (Capovilla et al., 2015).

The histone variant H2A.Z acts as a thermosensor for flowering time. H2A.Z is incorporated into nucleosomes by a chromatin-remodeling complex that includes ACTIN-RELATED PROTEIN6 (ARP6) and PHOTOPERIOD-INSENSITIVE EARLY FLOWERING1 (PIE1) (Talbert and Henikoff, 2014). H2A.Z incorporation into nucleosomes makes DNA less accessible for transcription factors and slows RNA polymerase II. This limits gene expression at lower temperatures because, with increasing temperature, H2A.Z nucleosomes are depleted (Talbert and Henikoff, 2014). Of relevance to flowering, H2A.Z occupancy at the *FT* promoter is decreased at higher temperatures, permitting promoter binding by PIF4 (Kumar and Wigge, 2010; Kumar et al., 2012). *PIF4*, initially identified as important in the shade avoidance response, was implicated in thermoresponsive flowering because the *pif4* mutant failed to accelerate flowering at elevated temperatures (Kumar et al., 2012). Similarly, *pif5* loss of function delays flowering at high temperature and the *pif4 pif5* double mutant flowered later than either single mutant, showing that both PIF4 and PIF5 accelerate flowering at elevated temperature (Fernández et al., 2016).

PHYTOCHROME INTERACTING FACTOR3, PIF4, and PIF5, but not PIF1 and PIF6, promote flowering when overexpressed in the phloem companion cells (Galvão et al., 2015). The PIFs promote flowering through induction of *FT* and its paralog *TWIN SISTER OF FT* (*TSF*) in response to warm nights and independently of *FT* during warm days (Thines et al., 2014; Galvão et al., 2015; Fernández et al., 2016). The increased expression of *FT* at high temperatures requires CO in addition to PIF4 and PIF5; *co pif4* double, and *co pif4 pif5* triple mutants flower later than *pif* single or double mutants. PIF4 and CO physically interact and this complex contributes to the induction of *FT* and *TSF* expression (Fernández et al., 2016). However, the *co pif4 pif5* triple mutant still flowers earlier at 27 than at 21°C (Fernández et al., 2016). Similarly, quadruple *pif1 pif3 pif4 pif5* (also called *pifQ*) loss of function mutants only partially suppress the early flowering and elevated *FT* expression at high temperature persists in the *arp6* mutant (Galvão et al., 2015). This indicates that the mechanism by which H2A.Z delays flowering must be more complex than simply through PIF interaction with the *FT* promoter and that there is additional complexity in the acceleration of flowering in response to elevated temperature.

SHORT VEGETATIVE PHASE plays a central role in thermoresponsive flowering as well as in vernalization. SVP encodes a flowering repressor and thermoresponsive flowering likely includes a reduction of SVP expression at higher temperatures, because SVP overexpression delays flowering at 27°C (Fernández et al., 2016) and *svp* loss of function mutants flower early and fail to modify their flowering time in response to temperature (Capovilla et al., 2015) (**Figure 1**). SVP forms repressor complexes with MADS box transcription factors related to FLC: FLM and MAF (Ratcliffe et al., 2003; Lee et al., 2013; Posé et al., 2013; Gu et al., 2013a). These complexes repress *FT* and *SOC1* transcription at low temperatures but decline in abundance at higher temperatures, relieving repression (**Figure 1**). The circadian clock imposes a circadian oscillation on SVP expression, linking thermosensitivity to circadian cycling (Fujiwara et al., 2008).

Gibberellic acid stimulates flowering. GA signaling entails the degradation of the DELLA transcriptional repressors; low GA levels allow the accumulation of the DELLAs, which delays flowering (Galvão et al., 2012; Yu et al., 2012). Inhibition of GA biosynthesis suppresses the acceleration of flowering at high temperature (Balasubramanian et al., 2006) and blocks the acceleration of flowering and increase of *FT* expression seen in the *arp6* mutant (Galvão et al., 2015). However, both *ft* and *ft tsf* mutants still accelerate flowering in response to exogenous active GA, indicating that GA can act independently of *FT* and *TSF*. Similarly, GA can accelerate flowering in the *arp6* mutant and the *pif3 pif4 pif5* triple mutant indicating that GA can act independently of H2A.Z incorporation and the *PIF* genes. Expression of a constitutively active DELLA protein at the shoot apical meristem (SAM) but not in the phloem companion cells prevented GA-induced flowering, indicating that the GA acts at the SAM, consistent with its independence from the *PIFs*, which induce *FT* and *TSF* in phloem companion cells. The action of GA at the SAM, at least in part, involves the induction of the floral inducers SPL3 and SPL5 (Galvão et al., 2012, 2015; Porri et al., 2012; Yu et al., 2012).

There is considerable natural variation in thermoresponsive flowering and *FLM* is a major-effect QTL (Balasubramanian et al., 2006). Consistent with FLM as a flowering repressor, the loss of function *flm-3* allele confers early flowering (Lee et al., 2013; Posé et al., 2013). The *FLM* primary transcript undergoes temperature dependent alternative splicing to yield two main isoforms that differ in terms of use of exon 2 (*FLM-*β) or exon 3 (*FLM-*δ); FLM-β binds DNA but FLM-δ does not (Lee et al., 2013; Posé et al., 2013). At lower temperatures the SVP-FLM-β complex is abundant and represses the floral integrators, *FT* and *SOC1*, but at higher temperatures the abundance of both FLM-β and SVP decreases, relieving repression (Lee et al., 2013; Posé et al., 2013). At higher (27°C) temperatures additional longer transcripts arise due to intron retention and the use of novel splice sites (Sureshkumar et al., 2016). Most of these longer transcripts include premature termination codons and are subjected to non-sense-mediated decay. The net result is a decreased abundance of the *FLM-*β transcript, the FLM-β isoform, and the SVP-FLM-β repressor complex (Sureshkumar et al., 2016).

A natural allele of *FLM*, in which a LINE retrotransposon has inserted into the first intron, confers early flowering both at 15 and at 21°C, although the effect was more pronounced at 15°C (Lutz et al., 2015). This insertion reduces abundance of both the major *FLM* transcripts, although temperature-dependent alternative splicing is preserved. Similar alleles were found in additional accessions, suggesting that this class of insertion confers early flowering at 15°C in summer annual accessions through reduced expression of the FLM-β isoform and the SVP-FLM-β repressor complex (Lutz et al., 2015).

SHORT VEGETATIVE PHASE also forms floral repressor complexes with MAF2, MAF3 and MAF4 (Ratcliffe et al., 2003; Gu et al., 2013a). *MAF2* has evolved a temperature dependent alternative splicing pattern independently from *FLM*. The abundant *MAF2* splice form at low temperatures encodes a functional MAF2 isoform that complexes with SVP to generate a floral repressor, but at elevated temperature an alternatively spliced intron-retaining variant encodes a prematurely truncated and non-functional MAF2 isoform that fails to repress flowering (Airoldi et al., 2015).

Temperature influences flowering, but the magnitude and direction of the temperature response depends on ecological details of the species under consideration (Capovilla et al., 2015). In *Boechera stricta*, a perennial relative of *Arabidopsis*, elevated temperature delays flowering (Anderson et al., 2011).

## Photoperiodic Flowering

In the photoperiodic pathway, the circadian clock regulates the induction of critical flowering inducers, CONSTANS (CO) and FT, via an external coincidence mechanism in which the external stimulus, light, must coincide with an inductive window that is restricted (gated) by the circadian clock (Romera-Branchat et al., 2014; Greenham and McClung, 2015; Song et al., 2015). The following simplification emphasizes several examples of external coincidence.

The circadian clock drives morning-specific expression of several *CYCLING DOF FACTOR* (*CDF*) genes whose protein products repress *CO* transcription. The CDF proteins are targeted for degradation by a SCF complex containing FLAVIN BINDING, KELCH REPEAT, F-BOX1 (FKF1), and GIGANTEA (GI) (**Figure 1**). Both FKF1 and GI exhibit circadian cycling in protein abundance. In short days, GI protein abundance peaks at dusk while FKF1 protein peaks after dark. This leads to the formation of the FKF1-GI complex in the dark. Thus, *CO* transcription is repressed until about dusk and *CO* mRNA accumulates after dusk. CO protein is unstable in the dark so, in short days, CO protein fails to accumulate and *FT* transcription is not induced.

In long days the phase of peak *GI* expression is delayed and coincides with that of FKF1 in late afternoon. FKF1 is a blue-light photoreceptor, and the interaction of FKF1 with GI is enhanced by blue light. This is a second example of external coincidence, when the peaks of FKF1 and GI proteins coincide in the light to allow the formation of the FKF1-GI complex to degrade the CDFs in the late afternoon. As a consequence, transcriptional repression of *CO* is relieved in the afternoon of long days and *CO* mRNA accumulates in the light, which permits the stabilization of nascent CO protein and activation of *FT* transcription.

*FLOWERING LOCUS T* transcription is also induced independently of CO. Several CRY2-INTERACTING bHLH (CIB) transcription factors accumulate in long days to stimulate *FT* transcription. The CIBs are activated in the afternoon by blue-light dependent interaction with CRY2 (Liu et al., 2008). In addition, CIB protein stability is enhanced via a blue light dependent interaction with the FKF1 relatives, ZEITLUPE (ZTL) and LOV KELCH PROTEIN 2 (LKP2), although not with FKF1 (Liu et al., 2013). Thus, this CO-independent induction of *FT* is mediated in the afternoon/evening of long days via two classes of blue light photoreceptors, CRY2 for CIB activation and ZTL/LKP2 for CIB stabilization, a third example of external coincidence.

## Conclusion

Studies of growth and flowering have emphasized the effects light intensity, quality, and duration (photoperiod), each of which has dramatic effects on vegetative morphology and the developmental transition from vegetative to reproductive growth (Arsovski et al., 2012; Casal, 2012; Song et al., 2015). Temperature also affects plant growth and reproduction, but only in recent years has it been realized that the effects of elevated but non-stressful temperatures on growth and reproduction can be of similar magnitude to those of light quantity and quality (Wigge, 2013; Quint et al., 2016). Temperature and light share some regulatory networks, but also employ specific regulatory pathways. One particularly prominent theme is the strong intersection of light and temperature signaling with time of day imposed by the circadian clock. In thermomorphogenesis as well as in thermosensitive and photoperiodic flowering, internal rhythms established by the circadian clock must coincide with the externally imposed environmental cycle of light and dark, an intersection termed “external coincidence.” Against a backdrop of global warming predicted to have major effects on the performance and distribution of wild species and strong adverse effects on crop yields (Willis et al., 2008; Wolkovich et al., 2012; McClung, 2014), it is crucial to integrate our understanding of temperature perception and response with other environmental responses, including biotic and abiotic stresses. Circadian clock function is intricately intertwined with each of these environmental response pathways (Greenham and McClung, 2015).

It is well-established that there is heterogeneity among plant species in terms of clock function and its relationship to flowering time (Song et al., 2015), so extrapolation to crops will require dedicated study in each species under consideration, using models established in *Arabidopsis* as guides.

## Author Contributions

All authors listed, have made substantial, direct and intellectual contribution to the work, and approved it for publication.

## Conflict of Interest Statement

The authors declare that the research was conducted in the absence of any commercial or financial relationships that could be construed as a potential conflict of interest.
